# Alcoholic hepatitis

**Published:** 2013-06-25

**Authors:** G Testino

**Affiliations:** Centro Alcologico Regionale – Regione Liguria, Alcohol Unit, Department of Internal and Specialist Medicine, IRCCS AOU San Martino-National Institute for Cancer research, Genova, Italy

**Keywords:** Alcoholic Hepatitis, Alcoholic Liver Disease, Liver Transplantation

## Abstract

Alcoholic hepatitis (AH) is a clinical syndrome characterized by jaundice and liver failure that generally occurs after decades of harmful alcohol consumption. Less severe forms of acute AH (AAH) frequently respond to alcoholic abstinence; whereas severe AAHs are characterized by a poor prognosis: up to 40-60% of these patients die within six months. Glucocorticoids currently remain the mainstay for treating severe AAH in patients with Maddrey’s Discriminant Function score > 32. Standard contraindications include recent upper gastrointestinal bleeding, renal insufficiency and uncontrolled infections. The evaluation of concomitant viral infections (hepatitis C and B viruses) is mandatory. Liver transplantation (LT), in non-responders patients, is a possible therapeutic option for severe AAH, but it is rarely used because a 6-month abstinence period is required before listing for LT. Unfortunately, most of these patients die before the end of this sober period. In our opinion, in case of severe AAH and in case of patients with a good social support and without severe psychotic or personality disorders, the lack of pre-LT abstinence period alone should not be considered a hindrance to LT.

## Introduction

Alcohol consumption is responsible for 3.8% of global mortality and 4.6% of disability-adjusted life-years (DALYs) lost due to premature death. The attributable burden in Europe, with 6.5% of all deaths and 11.6% of DALYs attributable to alcohol, is the highest proportion of the total ill health and premature deaths due to alcohol of all World Health Organization (WHO) regions. Europe shows particularly large sex differences in burden: the deaths attributable to alcohol being 11% and 1.8% for men and women, respectively. The young account for a disproportionate amount of this disease burden, with an alcohol-associated mortality of over 10% and 25% of female and male youth, respectively [**1**].

 Alcohol damages every organ and system in the body. Clinically, the most significant effects concern circulatory, nervous and hepato-gastroenterological systems.

 In the digestive tract, effects range from accelerated intestinal transit time and gastritis, leading to classical early morning nausea and diarrhoea, through to significant malabsorption and chronic pancreatitis.

 The association between chronic liver disease and alcohol consumption has been recognized for centuries. There is a spectrum of alcohol-induced hepatic injury, which ranges from minor biochemical and clinically insignificant damage, to fatty liver, alcoholic hepatitis and cirrhosis [**2,3**].

### Natural History

Alcohol consumption initiates liver damage via oxidative stress, endotoxin and inflammation [**4**].

In addition to ingestion via consumption of alcoholic beverages, small amounts of ethanol are also produced endogenously in normal intermediary metabolism and by microbial formation, especially in the gastrointestinal tract. The resulting concentrations in human venous blood are estimated to vary between 0-50n microns [**5**].

 The main alcohol metabolism occurs in the liver. There are three metabolic systems capable of carrying out ethanol oxidation in the liver: cytosolic alcohol dehydrogenase (ADH), the microsomal ethanol oxidizing system (MEOS) located in the smooth endoplasmic reticulum of hepatocytes, and catalase, located on peroxisomes. All these hepatic enzymes yield acetaldehyde as a product. 

 Alcohol dehydrogenase is the dominating enzyme pathway. 

 Acetaldehyde, produced by alcohol oxidation through any of the mechanisms outlined above, is rapidly metabolized to acetate, mainly by cytosolic Aldehyde Dehydrogenase 1 (ALDH1) and by mitochondrial Aldehyde Dehydrogenase 2 (ALDH2).

 Alcoholic liver disease (ALD) is believed to progress through histological stages: fatty liver (steatosis), steatohepatitis (alcoholic hepatitis, alcoholic steatonecrosis), fibrosis, cirrhosis and hepatocellular carcinoma (HCC).

 Alcoholic steatosis is mostly macrovesicular and more prominent in zone three of the liver acinus, which is the region surrounding the central veins. Pathophysiology of ALD has not been completely clarified yet. Ethanol metabolism alters the intra-mitochondrial redox potential via the generation of NADH by ADH. Therefore, ethanol causes oxidative stress. This impairs beta-oxidation of fatty acids and tricarboxylic acid cycle activity, resulting in elevated intra-hepatocellular free fatty acids, augmented formation of triacylglycerol, and increased rates of very low-density lipoprotein synthesis.

 There is also a non-oxidative pathway of ethanol. This involves the esterification of ethanol with fatty acids to form fatty acid ethyl esters, a reaction induced and catalysed by the enzyme fatty acid ethyl esters synthase. This pathway also generates phosphatidylethanol via phospholipase D with reduction of phosphatidylcholine [**2**].

 Acetaldehyde disrupts intestinal epithelial tight junctions increasing intestinal permeability to endotoxins. Endotoxemia plays a crucial role in hepatic damage by activating Kupffer cells to secrete a spectrum of cytokines and reactive oxygen intermediates, and by affecting hepatic sinusoids to enhance vascular permeability. These inflammatory mediators, such as TNF-alpha (together with an increase of TNF-alpha receptors), may contribute to further liver damage. It is well known that the elevation of TNF-alpha is associated with a worse prognosis.

 We can confirm that cellular oxidative-stress (caused by an imbalance between free radical generation and insufficient anti-oxidant defence mechanisms, including reduction in glutathione, phosphatidylcholine and vitamin E) in association with endotoxemia are the principal mediators for the progression to steatohepatitis and fibrosis.

 Approximately 20-40% of the subjects with steatosis have changes on liver biopsies consistent with steatohepatitis: steatosis plus hepatocellular injury with associated inflammation and fibrosis.

 A modification of intrahepatic gene expression has been evidenced in patients with alcoholic hepatitis in comparison to patients with only steatosis. In particular novel differentially expressed genes including claudins, osteopontin, CD209, selenoprotein and genes related to bile duct proliferation have been identified [**6,7**].

 Once steatohepatitis develops, hepatic morphology seldom returns to normal, even after abstinence, and the risk of developing cirrhosis increases. After the initial stellate cells activation (fibrogenesis), the hepatic stellate cells (HSC) respond to various stimuli in an autocrine and paracrine way to proliferate, migrate and contract, secreting extracellular matrix components, chemokine, cytokines, proteases and growth factors, as well as expressing signalling molecules and transcription factors.

 There are three main types of scarring which predominate in human ALD: centrilobular scarring, sinusoidal capillarization (pericellular fibrosis) and periportal fibrosis. The fibrosis process culminates in central-to-central veins bridging fibrosis and in regenerative nodules (cirrhosis). 

 Compensated cirrhotic patients, who continue to drink, show a five-year survival rate of about 70%; this rate increases to 90% if they abstain from further alcohol intake. In patients with decompensated cirrhosis, the five-year survival rate is of 50% in abstinent individuals, but drops to less than 30% in those who continue to drink [**2**].

 More recently, it has been evidenced how abstinence from alcohol at 1 month after diagnosis of cirrhosis was the most important factor determining survival with a 7-year survival of 72% for the abstinent patients versus 44% for the patients continuing to drink. Verrill et al. affirm that it is never too late to stop drinking, even with the most severe degrees of cirrhosis on biopsy [**8**].

### Is ALD progression reversible?

 Stopping alcohol consumption may completely reverse steatosis. It is known that advanced ALD prognosis is poor, with a survival rate of 23% at 5 years and of 7% at 10 years. An important issue is whether there is reversibility in the case of steatohepatitis/steatofibrosis: Diehl affirms that alcohol-induced steatohepatitis appears to be the “rate-limiting step" in the pathogenesis of alcohol-induced cirrhosis because almost 40% of the patients with the lesions develop cirrhosis within 5 years. Moreover, he declares that the evolution of more advanced stages of ALD depends on the balance between the degree of exposure to alcohol and the presence of other host attributes or confounding conditions [**2,9**].

 A past experience demonstrated how, in abstainer patients, the reversibility of alcoholic hepatitis is possible and it is confirmed by the return to an essentially normal architecture even in cases in which the alcoholic steatohepatitis was severe or was observed repeatedly for 3-5 years [**10**].

HSC are known to synthesize the excess matrix that characterizes liver fibrosis and cirrhosis. 

It is well known, in fact, that HSC play a pivotal role not only in fibrogenesis but also in fibrinolysis. Ethanol or acetaldehyde-derived oxidative stress promotes HSC production of extracellular matrix (ECM), mainly type I and type III collagen. Activated HSC express both the matrix-degrading enzymes (MMPs) and the tissue inhibitors of metalloproteinases (TIMPs). Failure of matrix degradation leads to ECM accumulation and progressive hepatic fibrosis.

 It is possible, in case of ethanol abstinence and in association with an exercise program, adequate nutrition and pharmacological treatment (mainly with antioxidant agents), to reverse the morphology of activated HSC to the quiescent phenotype, before irreversible fibrotic changes occur [**11,12**].

### Risk Factors

 The relationship between the quantity of alcohol ingested and the development of liver disease is not clearly linear.

 Women have been found to be twice as sensitive to alcohol-mediated hepatotoxicity and may develop more severe ALD at lower doses and with a shorter duration of alcohol consumption that men.

 O’Shea et al. [**13**] reported that the risk of developing cirrhosis becomes substantial with the consumption of 60-80 g/day of alcohol for 10 years or longer in men, and 20 g/day in women.

 Bellentani et al. evidenced that the odds of developing cirrhosis or lesser degrees of liver disease with a daily alcohol intake of > 30 g/day were of 13.7 and 23.6, respectively, when compared with non-drinkers [**14**]. 

 A more recent meta-analysis found increased risks of mortality from liver cirrhosis among men and women drinking 12-24 g of ethanol per day. Indeed, among women, a significant increase was also seen for those drinking up to 12 g/day. The European Association for the Study of Liver Disease suggests that, to date, if a threshold exists, it is very low, and difficultly detectable because of limitations in measuring consumption below 10-12 g per day [**1**].

 Drinking outside of meal times has been reported to increase the risk of ALD by 2.7-fold compared with those who consumed alcohol only at mealtimes.

 Binge drinking has also been shown to increase the risk of ALD and all-cause mortality [**15**].

There are important independent risk factors: visceral fat, obesity, hyperlipidaemia, diabetes, insulin resistance, metabolic syndrome, hepatitis viruses (mainly HBV e HCV) and Pearls grade. 

Raynard et al. [**16**] demonstrated that the Body Mass Index (BMI) and blood fasting glucose are independent risk factors for fibrosis in alcohol-induced liver disease. Pearls grade was also independently correlated with the fibrosis score. In particular, extra weight and obesity increase sensitivity to endotoxin liver injury. It is well known, in fact, that there is a close correlation between endotoxemia and the severity of alcohol-induced liver injury.

 Chronic hepatitis C infection and alcohol consumption account for 70-90% of all cases of chronic liver disease in the western world. In addition, up to 8-43% of patients affected by ALD are positive for anti-HCV.

 Alcohol consumption is an important risk factor in the histological and clinical progression of HCV infection. One recent study showed that the added effects of ethanol and HCV act synergistically to increase hepatic free radical formation and alteration of liver antioxidant defences; and that moderate alcohol consumption (< 50 g/ day) and heavy use (> 50 g/ day) increases the risk of developing oxidative stress 3-fold and 13-24 fold respectively [**17**]. Perlemuter et al. also demonstrated that HCV core protein expression and chronic alcohol consumption additively increase hepatic lipid peroxidation and synergistically increase hepatic TNF-α and TGF-β expression. TGF-β activates hepatic stellate cells (HSC) leading to an overproduction of extracellular matrix; TNF-α causes formation of ROS, which in turn may increase the hepatic fibrosis [**18**].

 There is evidence that the association between HCV infection and alcohol consumption favours fibrosis development independently to the daily ethanol dosage. It has been evidenced, in fact, a significant increase of fibrosis either under or over 80 g/ day.

 There are conflicting reports about the effects of alcohol on serum HCV-RNA. Some Authors have suggested that chronic ethanol consumption leads to a higher viral load; whereas the others have not found a relationship between HCV-RNA and the alcohol intake.

 Ongoing alcohol consumption has been reported to decrease the rate of response to antiviral therapy of hepatitis C. Patients should have been abstinent from alcohol for at least 6 up to 12 months before the initiation of Interferon (IFN) treatment. In our personal experience, there were fewer abstainers than drinkers among non-responders.

 More recently, it has emerged that patients affected by alcoholism, who were abstinent from 8-12 months, without psychotic or personality disorders, with a good social support and attending self-help groups had completed the treatment and had a response rate similar to the response of general population. Patients with a history of alcoholism should not be excluded from HCV therapy. Instead, additional support (cognitive behavioural therapy, frequency of self-help group, multidimensional family therapy) should be provided to these patients to ensure their ability to complete the therapy [**19**].

 In relation to HCC, a significant synergy between alcohol consumption (> 60-80 g/ day of ethanol), hepatitis virus infection and metabolic alteration has recently been demonstrated. Hassan et al. [**20**] have demonstrated a significant synergy between alcohol consumption, hepatitis virus infection and diabetes mellitus.

 In case of heavy alcohol consumption (> 80 g/ day) with chronic hepatitis virus infection (HBV or HCV) an OR of 53.9 (virus alone OR 19.1, alcohol alone 2.4) has been evidenced and in case of heavy alcohol consumption with diabetes (insulin-dependent, non-insulin-dependent) an OR of 9.9 (diabetes alone 2.4) has been evidenced.

 Recently, French et al. [**21**] have evidenced the role of the Toll-like receptor (TLR) signalling as the mechanism of liver stem cell/progenitor transformation to HCC. This mechanism explains how alcohol consumption increases the risk of HCC in hepatitis C, hepatitis B, diabetes, hemochromatosis, and alpha-1 antitrypsin deficiency. The synergism is due to an activation of a common pathway in which TLR signalling induces the production of both pro-inflammatory cytokines, through nuclear factor-kB activation, and growth factors, through activation of activator protein 1. In particular, hepatitis C virus-induced TLR is activated by endotoxemia associated with alcohol intake, leading to accentuated TLR signalling which in turn up-regulates the stem cell marker required for TLR-dependent liver oncogenesis [**22**].

Among host factors that could contribute to the variability of ALD, genetic determinants or so-called susceptibility genes have been most sought [**23**].

 Several genetic variations have been described: alcohol-metabolizing enzymes, genes related to oxidative stress, genes implicated in immune reactions, genes coding for fibrosis-associated factors and genes involved in the modulation of steatosis [**23**]. 

### Alcoholic Hepatitis: Clinical Features and Management

 Alcoholic hepatitis (AH) is a clinical syndrome of jaundice and liver failure that generally occurs after decades of heavy alcohol use [**24**]. 

 Patients with severe alcoholic hepatitis can have a clinical presentation almost similar to those with decompensated cirrhosis; and it may become difficult to establish whether they have associated cirrhosis. However, histologically, the majority of patients with severe alcoholic hepatitis have either significant fibrosis or cirrhosis of the liver.

 The average age of diagnosis is around 50 years old. The true incidence of AH is unclear. The prevalence of AH in patients who undergo liver biopsy is of about 20% and it may be present in as many as 10-35% of hospitalized alcoholic patients.

 Less severe forms (mild-moderate) of acute AH (AAH) frequently respond to alcoholic abstinence, whereas the prognosis of severe AAH is poor; up to 40% die within 6 months.

 In severe AAH, even in the absence of cirrhosis, the main vein that brings blood from the intestine and stomach into the liver (i.e., the portal vein) may come under increased pressure because of scarring of the liver, resulting in portal vein hypertension and complications [**2,25**].

 Decisions regarding the treatment are critically dependent on the ability to estimate a given patient’s prognosis. 

 In AH, the Maddrey discriminant function, a disease-specific prognostic score, has been used to stratify the patient's severity of illness. The initial formula was derived in the context of clinical trials of AH, and later modified to Maddrey discriminant function (MDF) = 4.6 (patient’s PT − control PT) + total bilirubin (mg/dl). A modified DF score >32 in the presence of hepatic encephalopathy predicts >50% mortality within 28 days in patients with alcoholic hepatitis. However, fatal outcomes have also been known to occur in patients with modified DF score <32, and this low specificity has suggested a need for alternative scoring systems.

 The Model for End-Stage Liver Disease (MELD) is a reliable measure of mortality risk in patients with end-stage liver disease. A MELD score ≥21 (within 24 hours of presentation) is a good predictor of 90-day mortality in patients with AH. MELD and modified DF scores (calculated within 24 hours of presentation) are equivalent in predicting 30- and 90-day mortality in patients with AH [**26**].

 The Glasgow Alcoholic Hepatitis Score (GAHS) is a composite scoring system based on age, serum bilirubin, blood urea nitrogen, PT, and peripheral leucocyte count. GASH equal or ≥ 9 is a predictor of mortality and is more accurate than DF in predicting both 28- and 84-day mortality but it is equivalent to MELD in predicting the 28-day mortality.

 The Lille model incorporates age, renal insufficiency, albumin, PT, bilirubin, and the evolution of bilirubin on day 7 to predict the 6-month mortality in patients with severe alcoholic hepatitis who have received corticosteroid therapy [**27**].

 From a pharmacological point of view, in association with lifestyle modification and appropriate nutrition, there is evidence to support the use of glucocorticoids, pentoxifylline (suppressor of TNF-alpha, prevents leukocyte adherence to vascular endothelium and down-regulates the expression of intracellular adhesion molecule-1 in monocytes), infliximab (a chimeric mouse/human antibody which binds to TNF-alpha), s-adenosyl-methionine (precursor of glutathione), antioxidants and colchicine.

 The presence of significant protein calorie malnutrition is a common finding in alcoholics, as are deficiencies in a number of vitamins and trace minerals, including vitamins A, D, thiamine, folate, pyridoxine, and zinc [2,13].

 Glucocorticoids currently remain the mainstay of treatment for severe alcoholic hepatitis. Nevertheless, the efficacy of corticosteroids is still considered as a controversial issue for some authors.

 A review of literature, made by Rongey et al. [**28**], supports a more discriminate use of glucocorticoids in patients with a Maddrey discriminant function score ≥ 32.

 Authors recommend stopping glucocorticoids if no improvement in bilirubin is seen after 7 days; switching to pentoxifylline is a reasonable alternative in that situation.

 Standard contraindications include recent upper gastrointestinal bleeding, renal insufficiency and uncontrolled infection.

 Christiansen and Gluud’s had performed a meta-analysis [**29**], obtaining data from more than 200 patients. Their results did not support the routine use of glucocorticoids in patients with alcoholic hepatitis, including those with encephalopathy.

 More recently, Mathurin and Lucey [**2**] have demonstrated improved survival with corticosteroid treatment. In their study, the patients allocated to corticosteroids treatment had higher 28-day survival than patients allocated to non-corticosteroids treatment: 80% versus 66%. The patients were classified as complete responders (Lille score < or equal 0.16; < or equal 35th percentile), partial responders (Lille score 0.16-0.56; 35th-70th percentile), and null responders (Lille > or equal; > or equal 70th percentile). This approach identified three patterns of responses, complete, partial, and null, with significant differences in survival benefit: 91% versus 79% versus 53%, p <0.0001. Corticosteroids showed a significant effect on 28-day survival in complete responders and in partial responders but not in null responders. 

 The management of portal hypertension with bleeding complicating ALD and AH is similar to that for other causes of end-stage chronic liver disease. In spontaneous bacterial peritonitis, hepatorenal syndrome (HRS) can be prevented by using oral pentoxifylline: in alcoholic hepatitis, it reduces the incidence of HRS and mortality to 8% and 24% vs. controls of 35% and 46% respectively.

 In severe end stage liver disease (with or without AH), hepatorenal syndrome (HRS) is a common complication that leads more than 90% of the patients to death within 3 months, unless they had been liver transplanted (LT). However, because of the limited survival of patients with HRS and the limited availability of organs, only a small percentage of patients can actually reach LT.

 Type-2 HRS is a moderate, steady renal impairment. It arises spontaneously and it is the main underlying mechanism of refractory ascites. Type-1 HRS is a rapidly progressive renal failure that is defined as doubling of serum creatinine reaching a level >2.5 mg/dl in less than two weeks. In type 1 HRS, a precipitating factor is frequently identified.

 In our experience, some suitable subjects awaiting LT, with type-2 HRS and RA, and non-responders to medical treatment, have been submitted to transjugular intrahepatic porto-systemic stent shunt (TIPS). The stent shunt was successfully established in all patients.

 As for the ascites, a complete response with total remission was obtained in 44.5% of the cases, while partial response was obtained in 55.5% of the cases. Regarding the renal functional parameters, a significant improvement after TIPS has been evidenced, even in alcoholic patients.

 In 8 patients affected by alcoholic advanced cirrhosis listed for LT, with type-1 HRS, RA and dilutional hyponatremia, TIPS has led to a good control of portal hypertension. 

 More recently, 9 patients affected by severe AAH and HRS have been submitted to TIPS. After TIPS, the renal function improved with a significant reduction in serum creatinine and an increase in urine volume. We can conclude that TIPS is a valuable option in patients with advanced alcoholic liver disease or severe AAH complicated by HRS, awaiting LT [**30-32**].

### Histological Features

 The histological features of alcohol induced hepatic injury vary, depending on the extent and stage of injury.

 These may include steatosis, lobular inflammation, periportal fibrosis, nuclear vacuolation, bile duct proliferation, fibrosis or cirrhosis.

 In the subset of patients with AH, a liver biopsy may demonstrate specific histological features, including confluent parenchymal necrosis, steatosis, deposition of intrasinusoidal and pericentral collagen, ballooning degeneration, and lobular inflammation affecting the perivenular regions in the earliest stages. 

 The liver may be infiltrated by polymorphonuclear cells, typically clustered around cytoplasmic structures known as Mallory bodies, which represent aggregated cytokeratin intermediate filaments and other proteins. The severity of inflammation (i.e., degree of polymorphonuclear leukocyte infiltration) and cholestatic changes correlate with poor prognosis, whereas the presence of megamitochondria may be associated with a milder form of AH. 

 Li et al. [**33**] have proposed a pathological diagnosis report of ALD, based on the evaluation of steatosis severity (F0-F4), inflammation severity (G0-G4) and fibrosis grading (S0-S4).

 Steatosis: F0, presence of fatty degeneration in < 5% of the hepatocytes; F1, presence of fatty degeneration in 5-30% of the hepatocytes; F2, presence of fatty degeneration in 31-50% of the hepatocytes; F3, presence of fatty degeneration in 51-75% of the hepatocytes; F4, presence of fatty degeneration in more than 75% of the hepatocytes. 

 Based on the severity of inflammation, alcoholic hepatitis is divided into four grades (G0–4): G0, no inflammation; G1, presence of a few balloon-shaped hepatocytes in acinar zone 3, sporadic isolated spotty acinar necrosis and peri-central vein inflammation; G2, presence of apparent balloon-shaped hepatocytes in acinar zone 3, more spotty acinar necrosis, Mallory bodies and mild to moderate inflammation of the portal area; G3, extensive balloon-shaped hepatocytes in acinar zone 3, pronounced spotty acinar necrosis, presence of Mallory bodies and apoptotic bodies, moderate inflammation of portal area or periportal inflammation, or both; G4, confluent necrosis or bridging necrosis, or both.

 Hepatic fibrosis is divided into four grades (S0–4): S0, no fibrosis; S1, focal or extensive perisinusoidal or pericellular fibrosis in acinar zone 3 and peri-central vein fibrosis; S2, fibrosis expanding to portal area, peri-central vein sclerosing hyaline necrosis, focal or extensive asterism-shaped fibrosis of portal area; S3, extensive fibrosis of acinar, focal or extensive bridging fibrosis; S4, cirrhosis [**33**].

### Alcoholic hepatitis and Liver Transplantation

 Liver transplantation (LT) for alcoholic liver disease has a favorable outcome, at least as much as that for other diagnoses, and better than that for HCV infection. 

 Liver transplantation is a possible therapeutic option for severe AAH, but it is rarely used because a 6-month abstinence period is required before the listing for LT. However, this period is arbitrary and it has never been demonstrated that it could affect the survival after LT. 

Even in cases in which there is evidence that shorter prelisting abstinence correlates with shorter time to first drink post-transplant [**36**], an optimal period of pre-transplant abstinence remains unclear.

The United Network for Organ Sharing and the French Consensus Conference affirmed that in case of end stage alcoholic liver disease, a duration of 6 months of abstinence before LT should no longer be the definite rule and should not be considered the determining factor for graft access [**37**].

 Since patients who do not recover within the first 3 months of abstinence are unlikely to survive, a shorter period (i.e. 3 months instead of 6 months) before LT seems to be more suitable [**38**].

 In case of severe AAH, a 6-month sobriety period is clearly inadequate. The high risk of early death in these patients makes the programming of liver transplantation be considered a therapeutic option.

 In our experience seven non-responder patients (median age: 49 years), with clinical evidence of severe AAH (MELD > 21 and DF > 32) and type-1 HRS, have been submitted to transjugular intrahepatic portosystemic stent shunt (TIPS); afterwards they have been successfully transplanted (30-45 days later). Steroid therapy is contraindicated in case of renal failure. HRS is a possible indication for TIPS placement, independently of MELD score.

 None of the patients relapsed after a period of 5 years [**34,35**].

 Mathurin et al. [**39**] selected 26 patients (median age 47.4 years) and placed them on the list for a liver transplant within a median of 13 days after nonresponse to medical therapy. The median time from listing to liver transplantation was of 9 days (95% CI). Non-response to steroids was defined as a Lille score of 0.45 or more or as a rapid worsening of the liver function. The 6-month survival rate was significantly higher among patients undergoing transplantation (77±8%) than among matched controls (23±8%, P<0.001). No alcoholic relapse was observed within the initial 6-month follow-up period. Three of 26 patients later resumed drinking alcohol. None of them had any graft dysfunction.

 Shawcross and O’Grady [**40**] underlined that a teenager who develops liver failure after a deliberate paracetamol overdose, after taking ecstasy, or after contracting hepatitis B through irresponsible sexual behaviour will have open access to LT. Why should his or her peer, with alcoholic hepatitis, be treated differently?

 A strict application of a period of sobriety as a policy for transplant eligibility is unfair to such patients, as most of them will have died prior to the end of the 6-month sober period.

 In our opinion, patients with severe AAH and decompensated liver disease should be listed for transplantation after a 3-months abstinence period. Determining factors for graft access might be the following: a good social support and the absence of psychotic or personality disorders.

 Post-LT patients, with limited comorbidities and a good social and familial support, should be offered an individual cognitive behavioral therapy; whereas patients with significant comorbidities and/or limited social support should have access to multicomponent programs (multidimensional family therapy, functional family therapy, brief strategic family therapy).

 Self-Help groups participation (the best known is Alcoholics Anonymous) should be mandatory.

 In our opinion, in patients with AAH not responding to medical therapy the lack of pre-LT abstinence alone should not be considered a barrier against being listed [**34,35,41,42**].
